# Molecular Mechanism for the Regulation of Microcystin Toxicity to Protein Phosphatase 1 by Glutathione Conjugation Pathway

**DOI:** 10.1155/2017/9676504

**Published:** 2017-02-27

**Authors:** Wansong Zong, Xiaoning Wang, Yonggang Du, Shuhan Zhang, Ying Zhang, Yue Teng

**Affiliations:** ^1^College of Geography and Environment, Shandong Normal University, 88 East Wenhua Road, Jinan, Shandong 250014, China; ^2^School of Environmental and Civil Engineering, Jiangnan University, 1800 Lihu Avenue, Wuxi, Jiangsu 214122, China

## Abstract

Glutathione (GSH) conjugation was an important pathway to regulate the toxicity of microcystins (MCs) targeted to protein phosphatases. To explore the specific molecular mechanism for GSH detoxification, two typical MC-GSHs (derived from MCLR and MCRR) were synthesized, prepared, and purified according to previous research. Then, the reduced inhibition effect for MC-GSHs on protein phosphatase 1 was verified by comparing with their original toxins. To further clarify the molecular mechanism for MC-GSHs detoxification, we evaluated the interactions between MCs/MC-GSHs and PP1 with the assistance of MOE molecule simulation. When GSH was introduced to MCs, the covalent binding (Mdha^7^ to Cys_273_), the hydrophobic interaction (Adda^5^ with PP1), the hydrogen bonds (especially for Lys^2^-Arg_96_ and Glu^6^-Tyr_272_), the covalent combination (between Mdha^7^ and Cys_273_), and the ion bonds (between Mn^2+^ and Asn_124_/His_248_/Asp_64_/His_66_) of MCLR/MCRR-PP1 complexes weakened to a certain extent, while the ion bonds between Mn^2+^ and His_173_/Asp_92_ residues increased. It was not difficult to find that the toxicity of MCs was closely related to the above sites/interactions and the above key information for MCs-PP1; MC-GSHs-PP1 complexes were important for clarifying the detoxification mechanism of MC-GSHs pathway. This study offers a comprehensive cognition on MCs toxicity regulation and provides valid theoretical support to control their potential risk.

## 1. Introduction

Microcystins (MCs) pose a worldwide health threat to humans and animals due to their increasing presence in aquatic environments as well as in water distribution systems [1, 2]. MCs are a class of hepatic heptapeptides produced by toxic cyanobacteria and posed a risk to environment when released as metabolic byproducts or during cyanobacteria cell lysis [3]. MCs shared the common structure of cyclo(-D-Ala^1^-L-X^2^-D-isoAsp^3^-L-Z^4^-Adda^5^-D-isoGlu^6^-N-methyldehydro-Ala^7^), in which X^2^ and Z^4^ were two variable amino acids, Adda^5^ was 3-amino-9-methoxy-2,6,8-trimethyl-10-phenyldeca-4,6-dienoic acid [4]. Due to the two variable amino acids and methylation/demethylation of other residues, there are more than 80 variants [5]. Among these toxins, MCLR and MCRR (L and R stand for variable amino acids Leu and Arg, resp.) are the most frequently found and studied variants [6, 7].

Toxicology experiments showed MCs had selectively hepatotoxicity through specific inhibition of protein phosphatases 1 (PP1) and 2A (PP2A), which in turn induced the hyperphosphorylation of some key control proteins in signal transduction [8]. The imbalance of protein phosphorylation/dephosphorylation promotes the oxidative damage of proteins and DNA, leading to cell structure disruption, apoptosis, liver necrosis, and intrahepatic hemorrhage [4, 7, 9]. In hepatic cells, MCs undergo a two-step interaction with PPs: the first step involves reversible binding that leads to rapid inhibition of catalytic activity; the second step involves formation of a covalent bond between the N-methyldehydroalanine residue (Mdha^7^) and a nucleophilic site on the PPs, leading to irreversible inactivation [10]. Crystal structure analysis of MCs-PP1/PP2A complexes confirms that MCs mainly attack the active site pocket of PP1/PP2A catalytic subunits through hydrogen bonds and ion bonds, and the hydrophobic cage structure adjacent to the active site pocket just can accept the hydrophobic side-chain of Adda^5^ [11].

The above features of MC-PP1/PP2A complexes may determine the typical inhibition effect of MCs on PP1/PP2A. Blocking or destroying their combination is important to regulate the inhibition effect of MCs on PP1/PP2A. Recent studies on MC regulation showed there is an enzymatic pathway for MCs detoxification via glutathione S-transferases (GSTs) [12–15]. The GST-derived metabolites of MCs are glutathione conjugates (MC-GSHs), which are obtained through the nucleophilic reaction of GSH thiol to the unsaturated carbonyl in Mdha^7^ of MCs [14, 16]. GSH conjugation appears to be the key step for MCs detoxication as MC-GSHs had lower toxicity and higher hydrophily compared with original toxins [17]. To date, the preparation and quantitative methods for MC-GSHs have been proposed, and the detoxification effect of GSH (with the aid of PPs inhibitory assays) has been widely studied. However, limited information on the structural features of MC-GSH-PPs complexes restricts the research on the interactions between MC-GSHs and PP1/PP2A. For this reason, the specific molecular mechanism for MC-GSH detoxification is not yet very clear. Thus, clarifying the molecular mechanism for the regulation of MCs toxicity (targeted to PPs) by MC-GSH pathway is of great importance and urgency.

To explore the detoxification mechanism of GSH to MCs toxicity, two primary MC-GSHs originated from MCLR and MCRR were synthesized through electrophilic addition reaction. After chromatography preparation and purification, their biological toxicity target to PP1 was evaluated and compared with that of MCLR and MCRR. To clarify the molecular mechanism for MC-GSH detoxification, we further evaluated the interactions between MCs/MC-GSHs and PP1 with the assistance of* Molecular Operating Environment* (MOE) software molecule simulation. MOE is an interactive, windows-based chemical computing and molecular modeling tool and can simulate the interaction between toxicant and protein. On the basis of toxicity evaluation and molecular simulation, the key action sites and interaction modes for the toxicity of MCLR/MCRR and MCLR-GSH/MCRR-GSH were identified and compared. Accordingly, the specific molecular mechanism for MCs toxicity regulation (by MC-GSHs pathway) was clarified.

## 2. Materials and Methods

### 2.1. Materials

MCLR and MCRR standards were purchased from Sigma (Saint-Quentin Fallavier, France). PP1 (1500 U/mL) from rabbit skeletal muscle were obtained from EMD Millipore (Darmstadt, Germany). HPLC grade acetonitrile, trifluoroacetic acid, and methanol were obtained from Merck (Darmstadt, Germany). Bovine serum albumin, dithiothreitol, GSH, MnCl_2_, p-nitrophenyl disodium orthophosphate, and tris(hydroxymethyl)aminomethane were purchased from Sinopharm (Shanghai, China).

### 2.2. Addition Reaction of GSH to MCs

In order to prepare MC-GSHs, 2 *μ*M MCLR/MCRR and 500 *μ*M GSH were mixed in 5% K_2_CO_3_ and incubated for 2 h at room temperature [18]. Then, the reaction mixtures were neutralized with 0.2 M HCl and applied to conditioned Cleanert C_18_ SPE cartridges (500 mg, Bonna-Agela) that were rinsed with 10 mL methanol and 15 mL water. The impurities were eluted with 10 mL 10% methanol and MCs/MC-GSHs were eluted with 10 mL 80% methanol. The eluted samples were evaporated to dryness in N_2_ flow and resuspended in 1 mL acetonitrile. The samples were stored in −20°C before HPLC and mass spectra (MS) analysis.

### 2.3. MCs and MC-GSHs Analysis

#### 2.3.1. Directed MS Analysis of MCs and MC-GSHs

The crude extracts for MCs and MC-GSHs were analyzed by a maXis UHR-TOF mass spectrometer (Bruker Daltonics). Samples were mixed with isometric acetonitrile (containing 0.1% trifluoroacetic acid) and injected into MS spectrometer with a syringe pump at 5 *μ*L/min. MS parameters were set as follows: positive ion mode, electrospray source voltage 4.2 kV, cone voltage 0.5 kV, desolvation gas N_2_ 0.5 bar, dry gas N_2_ 4 L/min, dry gas heater 180°C, and scan range 400–1500. Data acquisition was controlled with the* Compass software* and MCs/MC-GSHs could be detected according to their* m/z* signals.

#### 2.3.2. MS/MS Analysis of MCs and MC-GSHs

MC-GSHs were further identified by comparing their specific secondary ions with those of MCs standards. MC-GSHs were collected from LC separation at their specific retention times and injected into MS spectrometer with a syringe pump at 5 *μ*L/min. MS/MS parameters were set as Section 2.3.1 except that N_2_ collision gas was used and collision energies were adjusted at 50 eV.

### 2.4. MC-GSHs Preparation

Obtaining purified MC-GSHs was the precondition for biological toxicity evaluation. For this reason, resuspended samples containing MCs and MC-GSHs were further separated using a Great Eur-Asia C_18_ column (9.4 × 250 mm, 5 *μ*m, 120 Å) on the previously mentioned HPLC-MS system. Instrument parameters were set as in Section 2.3 except that the injection volume was 100 *μ*L and the elution rate was 2 mL/min. Subsequently, the purified MC-GSHs were collected manually according to their specific retention times, evaporated to dryness with N_2_, and dissolved in 200 *μ*L methanol. MS analysis of isolated MC-GSHs was performed to evaluate their concentrations and purity with MCLR/MCRR standards as references.

### 2.5. Protein Phosphatase Inhibition Assay for MCs and MC-GSHs

The biological toxicity of MCs and MC-GSHs was evaluated by a colorimetric protein phosphatase inhibition assay [19, 20]. Firstly, PP1 was diluted to 5 U/mL with a freshly prepared buffer of 50 mM tris(hydroxymethyl)aminomethane-HCl (pH 7.4), 2 mM dithiothreitol, 1 mM MnCl_2_, and 1 g/L bovine serum albumin. Then, 10 *μ*L PP1 was added to 100 *μ*L test samples in a 96-well polystyrene microplate. With gentle shaking, the microplate was kept at 25.0°C for a quarter-hour and p-nitrophenyl disodium orthophosphate was added. After 1 h, the absorbances of incubated samples (p-nitrophenol production) were measured in a THERMO/max microplate reader. The inhibition of test samples on PP1 could be expressed as follows:

(1) $$\begin{array}{c} I_{PP1}=\frac{\left(A_{control}-A_{sample}\right)}{A_{control}}\times 100\%, \end{array}$$

where *A*_control_ and *A*_sample_ were the absorbances of reference sample (without PP1) and test sample at 405 nm, respectively.

### 2.6. Molecular Simulation for the Interaction between PP1 and MCs/MC-GSHs

Molecular simulation calculations were performed with* MOE software (version number 14.09)*. The original structure for MCLR-PP1 complex was obtained from Protein Data Bank (PDB code 1FJM, http://www.rcsb.org/pdb/home/home.do). Models for MCLR and PP1 were extracted based on the structure of MCLR-PP1. Models for MCRR, MCLR-GSH, and MCRR-GSH were prepared based on the structure of MCLR. Before calculations, receptor PP1 was protonated by adding hydrogen atoms and small molecule ligands were minimized for energy optimization. Then, the interactions between toxins and PP1 were simulated (Amber 10: EHT, Solvation: R-Field) and the key parameters such as the total energies, total combination areas, hydrogen bonds, and ionic bonds for main interaction sites were obtained for clarifying the detoxification mechanism of MC-GSHs pathway. To keep the consistency of experiment conditions with PP1 inhibition assay, the experiment conditions for MOE simulation were set as follows: reaction temperature 25.0°C, pH 7.4, and salt 0.05 M.

## 3. Results and Discussion

### 3.1. MC-GSHs Synthesis and Identification

**Figure 1:**
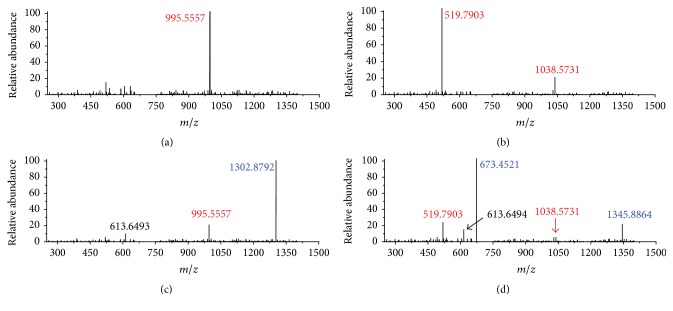
MS analysis for MCLR (a), MCRR (b), and the GSH electrophilic addition samples to prepare MCLR-GSH (c) and MCRR-GSH (d).

With conjugation with GSH, MCs might transform into specific MC-GSHs with different molecular weights which could be probed by mass spectrograph. For MCLR with a molecular weight of 994.5482, its primary MS signal was detected at* m/z* 995.5558 (Figure 1(a)), corresponding to the single-proton product of native toxin. For MCRR with a molecular weight of 1037.5652, two primary MS signals were detected at* m/z* 519.7903 and 1038.5731 (Figure 1(b)), corresponding to the double-proton and single-proton products. After electrophilic addition samples, MCLR and MCRR still exist (Figures 1(c) and 1(d)). However, they had lower intensities than the newly formed ions with MS signals at* m/z* 1302.8792 and 673.4521/1345.8864. As GSH was about 307.3235 Da, the above MS signals should be attributed to the addition products of GSH to MCLR or MCRR [21]. In addition, a product with MS signal at* m/z* 613.6493 was also found in both addition samples. This product should be attributed to the directed oxidation of sulfhydryl groups in two GSH, forming oxidized GSH (GSSG).

**Figure 2:**
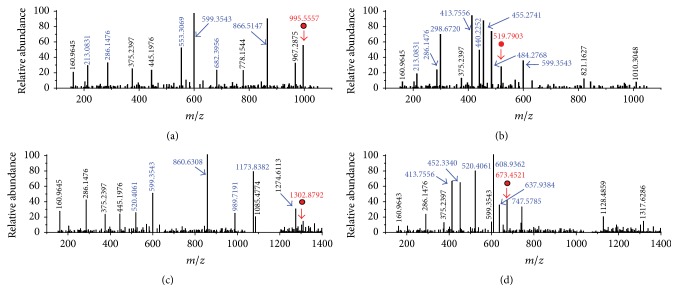
MS/MS analysis of MCLR (a), MCRR (b), and the identified electrophilic addition products MCLR-GSH (c) and MCRR-GSH (d). Conditions:* m/z* signals at 995.5557, 519.7903, 1302.8792, and 673.4521 correspond to the precursor ions of MCLR, MCRR, MCLR-GSH, and MCRR-GSH, respectively.

Molecular weight change could not provide further assistance for the identification of MC-GSHs. Accordingly, the specific generative mechanism of MC-GSHs was confirmed by comparing their secondary structures with MCLR and MCRR (with the assistance of* Compass Isotope Pattern* software). MS/MS analysis showed partial CID fragments of MCLR (*m/z* 995.5558) were detected at* m/z* 213.0831, 286.1477, 553.3069, 682.3956, and 866.5147 (Figure 2(a)), corresponding to the secondary structures of [Glu-Mdha+H]^+^, [MeAsp-Arg+H]^+^, [Mdha-Ala-Leu-MeAsp-Arg+H]^+^, [Arg-Adda-Glu-Mdha+H]^+^, and [Mdha-Ala-Leu-MeAsp-Arg-Adda+H]^+^/[Arg-Adda-Glu-Mdha-Ala-Leu+H]^+^ [22, 23]. For MCRR (*m*/*z* 519.7903, *z* = 2), its primary CID fragments were detected at* m/z* 213.0831, 286.1477, 298.6720, 413.7556, 440.2252, 455.2741, 484.2768, and 599.3552 (Figure 2(b)), corresponding to the ions of [Glu-Mdha+H]^+^, [MeAsp-Arg+H]^+^, [Mdha-Ala-Arg-MeAsp-Arg+2H]^2+^, [Ala-Arg-MeAsp-Arg-Adda+2H]^2+^, [Mdha-Ala-Arg-MeAsp+H]^+^, [Arg-Adda-Glu-Mdha-Ala-Arg+2H]^2+^, [Arg-MeAsp-Arg-Adda-Glu-Mdha+2H]^2+^, and [Arg-Adda-Glu+H]^+^/[MeAsp-Arg-Adda+H]^+^.

Based on the same strategy, the CID fragments of MCLR-GSH and MCRR-GSH could also be obtained. For MCLR-GSH with the* m/z* at 1302.8792 (Figure 2(c)), it had several identical fragment ions as that of MCLR (e.g., 160.9654 and 286.1477). In addition, MCLR-GSH also had partial new CID fragments at* m/z* 520.4061, 860.6308, 989.7195, and 1173.8082, corresponding to the ions of [Glu-Mdha+H]^+^+307.3230, [Mdha-Ala-Leu-MeAsp-Arg+H]^+^+307.3239, [Arg-Adda-Glu-Mdha+H]^+^+307.3239, and [Mdha-Ala-Leu-MeAsp-Arg-Adda+H]^+^/[Arg-Adda-Glu-Mdha-Ala-Leu+H]^+^+307.3235. It was not difficult to find that MCLR-GSH fragments containing Mdha^7^ were sustained by a 307.3235 ± 0.0005 Da difference with MCLR. In accordance with data in literature [24], these products should be from the additive reaction of GSH to the C=C bond in Mdha^7^ residual. MCRR-GSH (Figure 2(d)) (*m*/*z* = 673.4521) also had several identical fragments as that of MCRR except for [Glu-Mdha+H]^+^+307.3230, [Mdha-Ala-Arg-MeAsp-Arg+2H]^2+^+153.6619, [Mdha-Ala-Arg-MeAsp+H]^+^+307.3233, [Arg-Adda-Glu-Mdha-Ala-Arg+2H]^2+^+153.6621, and [Arg-MeAsp-Arg-Adda-Glu-Mdha+2H]^2+^+153.6616. Undoubtedly, MCRR-GSH fragments with Mdha^7^ residual were sustained by a 307.3235 ± 0.0005 Da difference with MCRR. According to the above analysis, GSH was undoubtedly added to the Mdha C=C bond of MCRR and formed MCRR-GSH.

### 3.2. Biological Toxicity Evaluation of MCs and MC-GSHs Target to PP1

To evaluate and compare the potential toxicity of MCLR, MCRR, and MC-GSHs to PP1, related MC-GSHs were prepared and purified with SPE and preparative chromatography techniques. The preparation and purification information for MC-GSHs were listed in Table 1. As MC-GSHs had higher concentrations (ranging from 1094 *μ*g/L to 1285 *μ*g/L) and higher purity (>98.3%), the prepared samples could be directed used to evaluated the toxicity of MC-GSHs.

**Figure 3:**
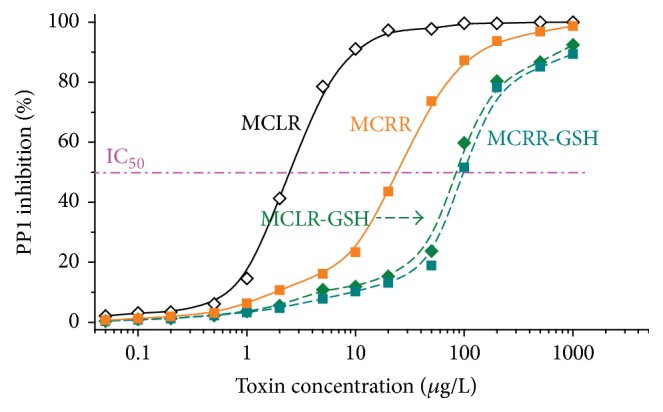
Inhibition curves for MCLR, MCRR, and related MCLR-DBPs on PP1. IC_50_ for MCLR, MCRR, MCLR-GSH, and MCRR-GSH on PP1 were about 2.5 ± 0.2 *μ*mol/L, 24.4 ± 0.5 *μ*mol/L, 86.6 ± 1.2 *μ*mol/L, and 98.7 ± 1.0 *μ*mol/L, respectively.

Based on PP1 inhibition experiment, the inhibition curves for MCLR, MCRR, and their conjugation products were plotted and their IC_50_ was calculated out.  Figure 3 showed MC-GSHs had lower toxicity than their native toxins in the sequence of MCLR (IC_50_ = 2.5 *μ*g/L) > MCRR (IC_50_ = 24.4 *μ*g/L) > MCLR-GSH (IC_50_ = 86.6 *μ*g/L) > MCRR-GSH (IC_50_ = 98.7 *μ*g/L). Similar to previous studies [17, 21], it was not difficult to find that GSH conjugation was an effective way to control the toxicity of MCs. Though the toxicity of MC-GSHs was obviously decreased, the secondary biotoxicity of MC-GSHs was real and nonnegligible. As a result, the secondary pollution of MC-GSHs also deserved further attention.

### 3.3. Molecular Mechanism for the Different Toxicity of MCs and MC-GSHS on PP1

**Figure 4:**
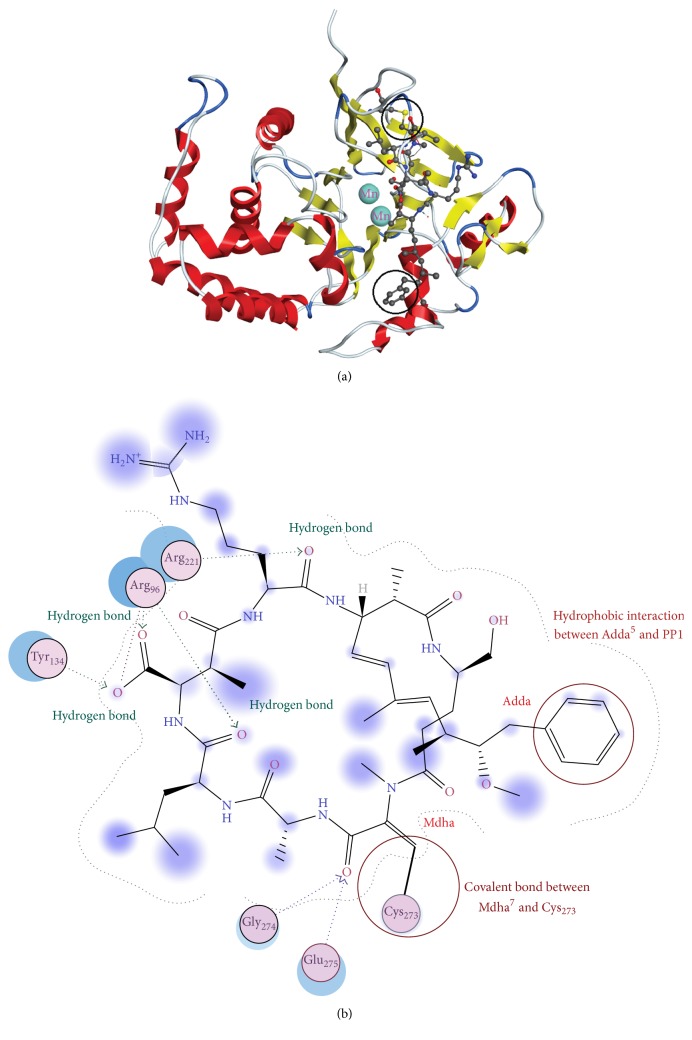
Molecular simulation results of MCLR and PP1 system. (a) The stereoscopic structure of MCLR-PP1 complex displayed in cartoon form. (b) The interaction between MCLR and related amino acid residues in PP1.

Although toxicity experiment revealed GSH conjunction had obvious regulation effect on MC toxicity, the detoxification mechanism has not been clarified as limited information was available on the structural features of MC-GSH-PP1 complexes. For these reasons, the specific interaction between MCLR, MCRR, MC-GSHs, and PP1 should be further explored with the assistance of molecular simulation. Between MCs and PP1, there were a reversible binding step through hydrogen bonding, ion bonding, hydrophobic interaction involving Adda^5^ residue, and an irreversible covalent bonding step involving Mdha^7^ residue and a nucleophilic site (with MCLR-PP1 complex serving as an example, Figure 4) [15, 25]. Accordingly, the total energies and total combination areas of toxin-PP1 complexes, the combination areas of Adda^5^ and Mdha^7^ residuals to PP1, the hydrogen bonds, and ionic bonds for main interaction sites were selected as the key parameters to assess the detoxification mechanism of GSH conjunction.

**Figure 5:**
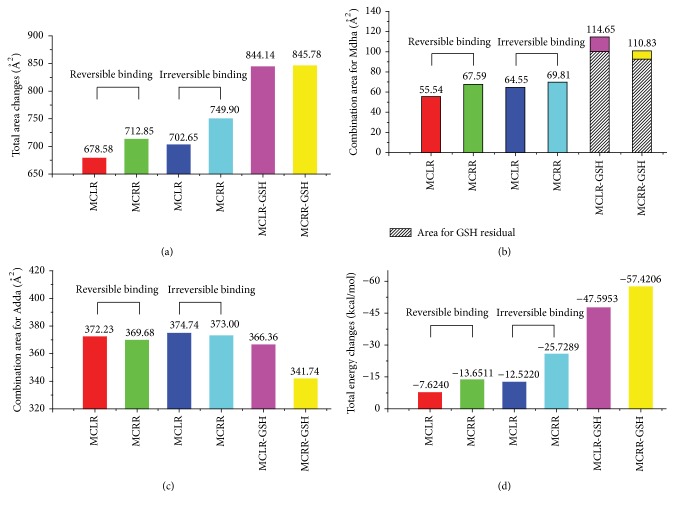
Molecular simulation results for the total combination area changes (a), the combination area changes for Mdha (b) and Adda (c) residuals, and the total energy changes (d) of target complexes.

Figure 5 showed the simulation information for the combination area and energy changes of toxin-PP1 complexes. Compared to the reversible binding step, the irreversible binding of MCLR/MCRR to PP1 both increased in total combination areas (Figure 5(a)), indicating that covalent binding of Mdha^7^ to Cys_273_ promoted the interactions between MCLR/MCRR and PP1. Though the specific covalent binding was destroyed by the introduction of GSH, the total combination areas still showed marked increasing tendency. In fact, the toxicity of MC-GSHs was much lower than their original toxins; the increments of combination areas should be attributed to the direct combination of GSH residue to PP1. The interpretation could be verified by the increased combination area for Mdha^7^/Mdha^7^-GSH with PP1: the combination areas increased after irreversible combination and significantly increased when GSH was introduced to MCs (Figure 5(b)). If the combination areas for Mdha^7^/Mdha^7^-GSH with PP1 were subtracted, the combination areas represented a marked slowdown. Accordingly, it could be ascertained that Mdha^7^ residue has certain relevance with MC toxicity. The toxicity of MCs to PP1 could be reduced by blocking the covalent binding of Mdha^7^ to PP1 by GSH conjunction pathway.

For Adda residue involved in hydrophobic interaction (Figure 5(c)), the combination areas for irreversible binding step were increased compared to reversible binding step. When GSH was introduced, the combination areas for Adda^5^ with PP1 represented marked slowdown. The combination areas for Adda^5^ with PP1 showed a positive correlation with the toxicity of MCs and their GSH conjunction products. Hydrophobic interaction for Adda^5^ with PP1 was an important factor for the toxicity of MCs and MC-GSHs. The toxicity of MCs to PP1 could also be reduced by blocking the hydrophobic interaction of Adda^5^ to PP1.

Compared to the reversible binding step, the irreversible binding of MCs to PP1 was more stable due to the significant decline in total energies (Figure 5(d)). Though GSH conjunction blocked up the combination of Mdha^7^ to Cys_273_ residue, the total energies for these complexes had more apparently downtrend. Actually, the conjunction products had much lower toxicity than original toxins and there were no direct relations between total energy changes and toxicity. The setback values for combination energies should be attributed to the extra interaction between GSH residues and PP1 as the increments of combination areas between MC-GSHs and PP1 weakened the surface energy of PP1.

**Figure 6:**
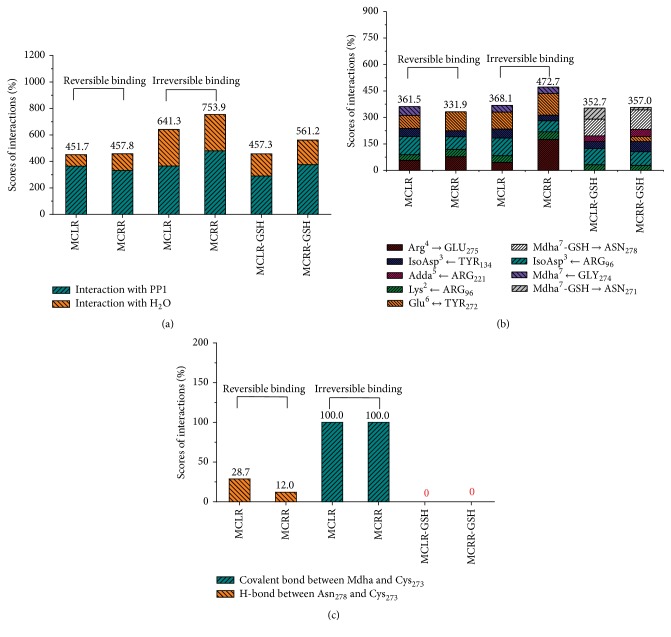
Scores for the total hydrogen bonds (a), the hydrogen bonds between primary interaction sites (b), and the specific covalent bonds between Mdha and Cys_273_ (c) of target complexes. Conditions: the total hydrogen bonds include the hydrogen bonds between MCs/MC-GSHs and H_2_O, MCs/MC-GSHs, and PP1 (Arg^4^-Glu_275_, Lys^2^-Arg_96_, IsoAsp^3^-Arg_96_, etc.).

Figure 6 showed the simulation information for hydrogen bonds and covalent bonds of toxin-PP1 complexes. Compared to the reversible binding step, the total hydrogen bonds (MCs/MC-GSHs with PP1 and H_2_O) for the irreversible binding step of MC-PP1 complexes were obviously promoted (Figure 6(a)). When GSH was introduced, the combination of toxins to PP1 was blocked up and the hydrogen bonds for toxin-PP1 complexes were dramatically decreased. The changing trends for total hydrogen bonds also could be applied to the hydrogen-bond change for toxin residues with PP1 or H_2_O. The positive correlation between hydrogen bonds and toxin toxicity showed hydrogen bonds also were important factors for the toxicity of MCs and MC-GSHs. As MCs and MC-GSHs had multiple potential hydrogen bonding sites to PP1, the hydrogen bond for single interaction site was obtained and illustrated in Figure 6(b). After irreversible binding, the hydrogen bonds for interaction sites Lys^2^ with Arg_96_ and Glu^6^ with Tyr_272_ were promoted, the hydrogen bonds for IsoAsp^3^ with Arg_96_ were decreased in some degree, and the hydrogen bonds for other interaction sites showed no consistent trends. Accordingly, the hydrogen bonds for interaction sites Lys^2^-Arg_96_ and Glu^6^-Tyr_272_ were closely related to the toxicity of MCs and MC-GSHs. Although three new types of hydrogen bonds for interaction sites Adda^5^ with Arg_221_, Mdha^7^-GSH with Asn_278_, and Mdha^7^-GSH with Asn_271_ were formed after GSH conjugation, the hydrogen bonds for interaction sites Arg^4^ with Glu_275_, Lys^2^ with Arg_96_, Glu^6^ with Tyr_272_, and Mdha^7^ with Gly_274_ still represented marked slowdown. Accordingly, the regulation effect of GSH for MCs toxicity was closely related to the above interaction sites. The toxicity of MCs to PP1 could be controlled by enhancing the hydrogen bonds for interaction sites Lys^2^ with Arg_96_ and Glu^6^ with Tyr_272_ and by blocking the hydrogen bonds for interaction sites IsoAsp^3^ with Arg_96_, Arg^4^ with Glu_275_, Lys^2^ with Arg_96_, Glu^6^ with Tyr_272_, and Mdha^7^ with Gly_274_.

As the irreversible binding step between MCs and PP1 involved the nucleophilic site Cys_273_, data for the interactions of toxins with residue Cys_273_ were also obtained (Figure 6(c)). For the reversible binding step of MCs to PP1, Cys_273_ had no direct interaction with Mdha but combined with Asn_278_ residue by hydrogen bonds. For the irreversible binding step, the formation of covalent bonds between Cys_273_ and Mdha^7^ should overcome the hydrogen bonds between Cys_273_ and Asn_278_. When GSH was introduced, the interactions between Cys_273_ and Mdha/Asn_278_ were destroyed. Considering the inhibition effect of MCs and MC-GSHs on PP1, the covalent combination of Mdha residue and Cys_273_ had certain relevance with the toxicity of MCs and MC derivatives.

**Figure 7:**
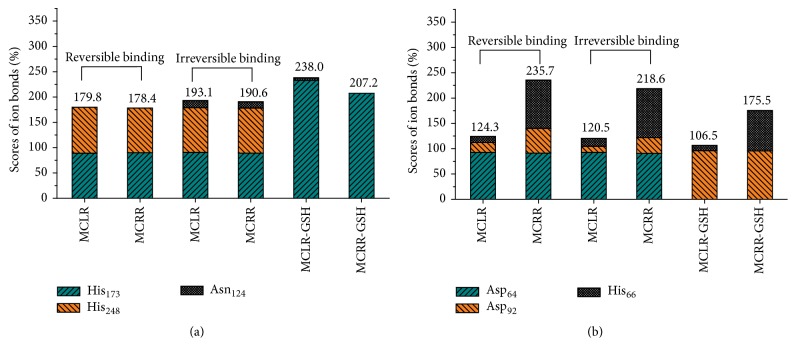
Scores for the major ion bonds involved in the interactions with Mn^2+^ ions in catalytic center of target complexes.

As PP1 was a type of metalloenzyme and regulated by two Mn^2+^ ions, the discrepant inhibition effect of MCs and MC-GSHs on PP1 might be mediated by Mn^2+^ ions. Specific interactions involving Mn^2+^ ions in PP1 catalytic center were also investigated. For the first Mn^2+^ ion (Figure 7(a)), the total ion bonds were promoted with irreversible binding of MCs with PP1 (mainly attributed to the new ion bond between Mn^2+^ and Asn_124_). When GSH was introduced, the ion bond between Mn^2+^ and His_248_ was totally destroyed. However, due to the significantly increased ion bond between Mn^2+^ and His_173_, the ion bonds for MC-GSH-PP1 complexes are still enhanced to a certain extent. Accordingly, the interaction for Mn^2+^ and His_173_ residue was positively correlated with the activity of PP1; the interactions for Mn^2+^ and Asn_124_/His_248_ residues were negatively correlated with the activity of PP1. For the second Mn^2+^ ion (Figure 7(b)), its ion bond with Asp_64_ remained constant but its ion bond with His_66_ increased when MCLR/MCRR irreversibly bind to PP1. However, due to the decreased ion bond between Mn^2+^ and Asp_92_, the total ion bonds showed downtrend. When GSH was introduced, the ion bond between Mn^2+^ and Asp_64_ was totally destroy; the ion bond between Mn^2+^ and His_66_ was weakened. Though the interaction between Mn^2+^ and Asp_92_ was dramatically enhanced, the total ion bonds were still weakened. Accordingly, the interaction for Mn^2+^ and Asp_92_ residue was positively correlated with the activity of PP1; the interactions for Mn^2+^ and Asp_64_/His_66_ residues were negatively correlated with the activity of PP1. The toxicity of MCs to PP1 also could be controlled by enhancing or reducing the specific ion bonds between Mn^2+^ and related sites.

## 4. Conclusions

Aiming at clarifying the detoxification mechanism of GSH conjugation pathway for the toxicity of MCs target to PPs, tow typical GSH conjugation products (MCLR-GSH/MCRR-GSH) were prepared, separated, and purified. According to PP1 inhibition experiment, MCLR-GSH/MCRR-GSH showed evident control effect on the toxicity of MCs. Based on molecular simulation, the specific regulation mechanism of GSH conjugation pathway was clarified: data for combination area ascertained that the toxicity of MCs was controlled by enhancing the covalent binding of Mdha^7^/Mdha^7^-GSH to PP1, the hydrophobic interaction between Adda^5^ with PP1; data for combination energy showed the extra decreased trends for MC-GSHs with PP1 merely attributed to the weakened surface energy; data also showed the toxicity of MCs was controlled by enhancing the hydrogen bonds (especially for interaction sites Lys^2^-Arg_96_ and Glu^6^-Tyr_272_) and the covalent combination (between Mdha^7^ and Cys_273_). Specific investigation on the interactions involving Mn^2+^ ions in catalytic center also showed GSH conjugation promoted their interactions with His_173_/Asp_92_ residues but weakened the interactions with Asn_124_/His_248_/Asp_64_/His_66_ residues.

## Figures and Tables

**Table 1:** Preparation and purification information for MCLR-GSH and MCRR-GSH.

Conjugation products	Eluted time^a^	Concentration	Total volume	Purity^c^
MCLR-GSH	12.54 min	≈1285 *μ*mol/L^b^	10*∗*100 *μ*L	98.3%
MCRR-GSH	8.43 min	≈1094 *μ*mol/L	10*∗*100 *μ*L	98.7%

a: collection time was set for 0.5 min (±0.25 min around the eluted time).

b: with 200 *μ*mol/L MCLR (MCRR) serving as the inner standard for quantification and assuming MCLR and MCLR-GSH (MCRR and MCRR-GSH) had approximate protonated efficiencies.

c: purity was directed calculated by MS signals and defined as MC-GSH/(MC + MC-GSH)*∗*100%.
